# The impact of China’s energy saving and emission reduction demonstration city policy on urban green technology innovation

**DOI:** 10.1038/s41598-023-42520-4

**Published:** 2023-09-13

**Authors:** Changfei Nie, Ruyi Li, Yuan Feng, Zhi Chen

**Affiliations:** 1 School of Economics and Management, Nanchang University, Nanchang, China; 2 Jiluan Academy, Nanchang University, Nanchang, China; 3 College of City Construction, Jiangxi Normal University, Nanchang, China; 4 School of Economics and Trade, Hubei University of Economics, Wuhan, China

**Keywords:** Environmental economics, Sustainability

## Abstract

Urban green technology innovation (UGTI) is strongly tied to environmental regulations, which can successfully balance economic and environmental benefits. Selecting the panel data for 280 Chinese cities during 2006–2019, we take the energy saving and emission reduction (ESER) demonstration city policy as a quasi-natural experiment, then employ the difference-in-differences model to examine the effect and its mechanisms of ESER policy on UGTI. Empirical results show that the ESER policy can significantly promote UGTI, especially in the western region, the northern region, and cities with weak government environmental attention. At the same time, China’s ESER policy has a stronger promoting effect on UGTI in cities where environmental targets are more stringent. Mechanism analysis shows that the policy mainly promotes UGTI through two channels: increasing the proportion of science and technology expenditure in fiscal expenditure and upgrading the structure of the industry. In addition, we find that the development of UGTI has positive environmental effects by lowering carbon emissions and air pollution. The findings not only enrich the literature on environmental regulation policies and UGTI at the theoretical level, but also provide references for policymakers to specific implementation methods in further enforcing environmental regulation policies to improve UGTI.

## Introduction

Green technology innovation has emerged globally in recent years as the idea of sustainable development has gained momentum, and it is now at the center of nations' ecological development policies^1^. Green technology innovation may successfully balance economic and environmental benefits, improve the effectiveness of the green economy^2^, and boost high-quality economic development by fusing green and innovation^3^. Cities are centers of human activity and major carriers of economic and social development. Statistics show that cities consume 75% of the world's energy and emit 80% greenhouse gases^4^. Therefore, an important question in the field of environmental economics is how to effectively encourage UGTI activities and improve the level of UGTI.

At the same time, in order to effectively respond to climate change and environmental pollution problems, countries such as the United States, Japan, and South Korea have launched a series of ESER policies through legislative or administrative means^5^. As the largest developing country and the highest carbon emitter, China is under tremendous pressure to save energy and reduce emissions. To explore the efficient route of ESER in cities and build replicable and cutting-edge experiences in ESER through pilot trials, the Chinese government implemented the ESER demonstration city policy. In 2011, the policy was put into effect, with special emphasis on promoting ESER through GTI. As a result, it is reasonable to assume that this legislation will have a significant effect on UGTI.

In view of this, our study uses panel and urban green patent data of 280 Chinese cities during 2006–2019, take the pilot policy of ESER demonstration city as a quasi-natural experiment, and use the difference-in-differences (DID) model to test the policy's impact effect and mechanism on UGTI. We find that the ESER policy significantly improves UGTI, increasing the per-capita number of invention and green utility model patent applications by approximately 6.50% and 10.07%, respectively. According to the mechanism analysis, the ESER policy encourages UGTI through two channels: increasing the proportion of science and technology spending in fiscal expenditures and upgrading the industrial structure. The heterogeneity analysis shows that the promotion effect is greater the western region, the northern region, and cities with weak government environmental attention. At the same time, China’s ESER policy has a stronger promoting effect on UGTI in cities where environmental targets are more stringent. Further, we analyze the environmental benefits, demonstrating that UGTI can effectively cut carbon emissions and air pollution, which can achieve synergistic carbon and pollution reduction.

Compared to other papers, this study makes three possible marginal contributions. First, From the research perspective, while existing literature investigates the causality between environmental regulation policy and green innovation^6,7^, few studies specifically examine the impact of the ESER policy on UGTI. In the few literatures on the assessment of the effects of the ESER policy, other academics mainly focus on the policy's impact on carbon emissions^8^, sustainable development^9^, and other factors without considering how the policy affect UGTI. This study assesses the impact of Porter's hypothesis on UGTI from the perspective of the ESER policy and is a useful contribution to the existing literature, providing the latest empirical evidence for Porter’s hypothesis. Second, in terms of research contents, our study examines the mechanisms in two ways: fiscal effects and structural effects, and further examines the heterogeneity of its impact on the degree of UGTI in different cities. In addition, we further examine the environmental benefits of UGTI from the perspective of the synergistic effect of reducing carbon emissions and air pollution, which provides empirical evidence for a comprehensive and systematic understanding of the implementation effects of ESER policy. Third, in terms of the research's significance, the study not only contributes to the scientific formulation of ESER policies and the effective promotion of UGTI, but also provides a basis for policymakers to utilize fiscal means to improve UGTI levels.

The reminder of the paper is structured as follows: the literature review is in Section “Literature review”, the background of the ESER demonstration policy is reviewed and the theoretical hypothesis is put forth in Section “Policy background and theoretical hypothesis”. The identification approach, variables, and data for this investigation are presented in Section “Research design”. The empirical findings and robustness tests are presented in Section “Empirical results and analysis”. The mechanism test, heterogeneity analysis, the impact of the Eleventh Five-Year Plan and environmental effects of UGTI are covered in Section “Further analysis”. The conclusions and associated policy implications are presented in Section “Conclusions and policy implications”.

## Literature review

In substance, the ESER policy is a form of environmental regulations, so this paper is mainly closely related to the literature on environmental regulation and UGTI. In general, the existing environmental regulation literature has comprehensively analyzed the relationship between environmental regulation and green innovation at the national^10^, provincial^11^, prefecture-level city^12^, industrial^13^, and enterprise levels^14^, respectively. However, on the relationship between environmental regulation and UGTI, the literature has not formed a unified view.

The first perspective suggests that environmental regulation has a significant positive effect on UGTI. Chen et al.^15^ used the panel data of 281 cities in China during 2004–2016 with the fixed-effect regression model to test and found that environmental regulation can promote UGTI. Different academics have different views on the regional heterogeneity of environmental regulation for promoting UGTI. Li et al.^16^ found that the effect of UGTI in the central and western regions is more significant than that in the eastern region. Using panel data of 152 cities in China from 2005 to 2019, Qiu^17^ found that environmental regulation had a more positive impact on UGTI in cities with higher initial innovation conditions. However, Zhang et al.^18^ found that UGTI in central China and resource-based cities had a more significant promoting effect. Using panel data of 41 cities in the Yangtze River Delta city cluster, Wang et al.^19^ and Zhou et al.^20^ discovered that there is a spatial spillover effect of environmental regulation on UGTI. Wang discovered that UGTI in the Yangtze River Delta cluster shows a trend of high in the east and low in the west, while Zhou found that the expansion of cities reduces the innovation capacity of cities.

The second perspective suggests that environmental regulation has no impact or even has a inhibitive impact on the development of UGTI. Considering the Low Carbon Pilot Cities (LCPC) program in China from 2004 to 2016 as a quasi-natural experiment, Tian et al. found that environmental regulation has no effect on UGTI. Based on data of 103 cities in China from 2007 to 2016, Nie et al.^21^ concluded that the impact of environmental regulation on UGTI is uncertain and found no evidence of a regional Porter effect stimulated by environmental regulation. Zhou et al.^22^ used the Yangtze River Delta in China as an example and adopted the spatial econometric model of economic matrix and comprehensive correlation matrix. They discovered that through the industrial structure effect and spillover effect, the spatial self-selection of environmental regulation somewhat dampens the enthusiasm of UGTI, Yang and Wang^23^ came to the same conclusion as Zhou. Environmental laws have also been demonstrated to conditionally encourage UGTI in the literature. If foreign investment and environmental restrictions are not coordinated, environmental rules may hinder UGTI^24^. According to Luo et al.^25^, market environmental regulations and foreign direct investment have a negative impact on UGTI, whereas command-and-control and informal environmental regulations have a significant Porter effect on UGTI.

The third view is that there is a non-linear relationship between environmental regulation and UGTI. Lanoie et al.^26^ suggested that the relationship between environmental regulation and UGTI is non-linear, with coefficients varying with the range of regulation intensity. Based on data of 105 Chinese cities, Du et al.^27^ utilized a partial linear functional coefficient panel model and discovered that the influence of environmental restrictions on the growth of UGTI is influenced by the level of urban economic development. Fan et al.^28^ created a geographical measurement model based on a geographic weight matrix using data of 235 Chinese cities from 2004 to 2016. They discovered a positive U-shaped association, which is in line with Ouyang et al.^29^ Zhang used data from Xi'an, China, from 2003 to 2016 and found that market-based regulations are more effective than command-and-control environmental regulations in promoting UGTI. Guo^30^ and Zhang et al.^31^ argued that the relationship between environmental regulation and UGTI is an inverted U-shaped relationship. According to Yang^32^, environmental regulation has a significant V-shaped threshold effect on UGTI, and at different stages, the intensity of environmental regulation has varying relative intensities on the follow-cost and reverse effect of UGTI.

There are fewer pertinent studies that concentrate on the ESER policy. Using panel data of 284 cities from 2013 to 2018, Wang and Qiu^33^ found that the ESER policy could effectively reduce environmental pollution, and it had a more significant emission reduction effect in cities with more abundant human capital and higher financial expenditure levels. Further research by Lin and Zhu^9^ revealed that the ESER’s emission-reduction benefit only lasts for the duration of the demonstration phase. Meanwhile, both Xu et al.^34^ and Du and Wang^8^ found that the ESER policy is effective in reducing urban carbon emissions. ESER is more effective at reducing carbon emissions in eastern and western, non-mineral resources, non-old industrial areas, and cities with high financial self-sufficiency, according to the research by Xu. However, Du and Wang discovered that the ESER policy works better in cities with greater employment to urban GDP ratios. Additionally, Lin and Zhu^35^ used the NDDF method to create an eco-efficiency factor to further investigate the impact of the ESER policy on sustainable urban development, and found that the ESER policy effect is ineffective in the short term, and the policy effect started to appear after three years of implementation.

From the above reviews, we can conclude that while the literature has more thoroughly investigated the causal relationship between environmental regulation and UGTI, it has not yet come to a consensus. A small number of recent studies have concentrated on the effects of the ESER policy, but even fewer studies particularly look at the effects of the ESER policy on UGTI. In an effort to further research in adjacent domains, we strive to view this as a significant development.

## Policy background and theoretical hypothesis

### The ESER demonstration city policy

The 11th Five-Year Plan in China is where ESER got its start. According to the proposal, the total emissions of key pollutants will be decreased by 10%, and the amount of energy saved per unit of GDP will be lowered by approximately 20%. The National Development and Reform Commission implemented the ESER policy in 2011 in an effort to reduce energy consumption and pollutant emissions, support economic structure adjustment, and change the mode of economic development. The policy designated eight cities as the initial group of ESER demonstration cities, including Beijing and Shenzhen. Ten and twelve ESER demonstration cities, were formed in 2013 and 2014, respectively, and the strategy is spreading. The Chinese government hoped that the demonstration cities, supported by the policy, will set an example and play a leading role in energy conservation and emission reduction, thus laying the foundation for the promotion of the policy throughout the country. In order to effectively mobilize local governments and ensure effective policy implementation, the National Development and Reform Commission has established a special reward and punishment mechanism. The workload, the impact of ESER, and development of long-term procedures were the key areas of focus in the performance assessment. Specifically, demonstration cities that received great ratings will receive 20% of the funding from the central government; demonstration cities that received qualified ratings will not receive any compensation; and demonstration cities that received unqualified ratings will lose 20% of the cash. It can be expected that the policy will have a significant impact on UGTI activities with the encouragement and constraint of a strict reward and punishment mechanism.

### Theoretical hypothesis

#### ESER demonstration city and urban green innovation

The ESER policy has a both incentive and constraint effect on UGTI activities. In terms of incentive policy, the policy increases the government’s fiscal revenue, which can be used by the government to give financial subsidies or tax concessions to enterprises with high pollution and energy consumption. Such concessions and subsidies have a direct impact on alleviating the paucity of green technology innovation resources and financing constraints in enterprises while stimulating and enhancing their green technology innovation activities^36^. Multiple studies also validate the positive impact of financial subsidies in promoting green technology innovation in enterprises^37^. For constraining effects, the ESER demonstration policy’s stringent environmental regulations will subject companies to pressure; however, it is vital to note that the Porter hypothesis purports that suitable environmental regulations can lead to increased innovation activities by companies, helping offset environmental protection costs and create a more competitive marketplace^38^. Numerous studies indicate that environmental regulatory policies facilitate the promotion of green technology innovation^39^.

At the same time, in practice, the ESER policy places a strong emphasis on UGTI, which is reflected in the various deployments made by demonstration cities. For instance, Changsha prioritizes the support of key enterprises in implementing energy saving technology transformation, as well as promoting the use of advanced energy-saving and environmental technologies and equipment while enhancing the green technology innovation level of such enterprises. Baotou invests a total of 19.4 billion in the implementation of 158 green innovation projects, including green technology innovation, desulfurization and denitrification, zero-emission sewage, and centralized coal combustion treatment for steel and electric power enterprises. Meanwhile, Hebi City, focuses on technology innovation in coal-fired power, cement, and chemical plants with large emissions and high energy consumption, emphasizing the upgrading of coal-fired power generation units. Based on these findings, we propose the following hypothesis:

##### Hypothesis 1

The ESER policy can reduce UGTI.

#### Mechanisms of ESER promoting UGTI

After reviewing the literature and considering the background of the ESER policy, we believe that the policy can promote UGTI through two channels: fiscal effect and structural effect.

First, the fiscal effect. On the one hand, technology innovation is an important driving force for green development, which cannot be separated from the necessary financial support, especially government financial input. As China's economy focuses more and more on high-quality development and ecological civilization construction, the government's expectations for environmental protection and green development are getting higher and higher. Finance will increase R&D investment in green and low-carbon areas, with financial expenditure on science and technology becoming an important driver of green technology innovation. Due to the complexity of the green innovation process, there is little incentive for enterprises to carry out green innovation activities without government involvement and financial support. More attention to science and technology investment by the central government can reduce the cost and risk of enterprise innovation, enhance the enthusiasm of enterprises for green technology innovation and gather talents^40,41^, thus increasing UGTI. Lin and Zhu^42^ find that China's financial R&D expenditure can produce obvious technological effects and promote the development of the green economy. It is proved that ESER demonstration cities, after receiving central financial subsidies, are asked to increase the restructuring of their fiscal expenditures to provide direct financial support for green innovation and other science, technology and innovation activities.

On the other hand, government R&D subsidies have an impact on green technology innovation, which has been confirmed by many studies. Government R&D expenditures can reduce innovation costs and risks for firms, provide the necessary policy subsidies for innovation activities with long investment cycles, and increase the incentives for green innovation in high energy-consuming firms. Qi et al.^43^ find that government subsidies and R&D investments improve energy transition performance as well as promote green technology innovation.

An excellent illustration of the positive impacts the ESER policy can have on scientific and technological innovation is seen in the subsidy funds granted to Baotou, Xuzhou, and Nanning City by the central government, each 1.2 billion yuan. For instance, Baotou City directs 19.4 billion yuan toward technology innovation within its iron and steel and electric power enterprises, while Xuzhou City invests 34 billion yuan in 107 demonstrative projects designed to advance GTI and reduce energy emissions. Similarly, Nanning City invests 13.838 billion yuan toward realizing GTI targets in its industrial enterprises. Therefore, the ESER policy can increase the government's financial expenditure on science and technology, which will promote the development of UGTI. For this reason, we propose the following hypothesis:

##### Hypothesis 2a

The ESER policy can induce UGTI by increasing fiscal expenditures on science and technology.

Second, the structural effect. This is achieved by eliminating backward production capacity in ESER demonstration cities, cultivating strategic emerging industries and modern service industries, as well as optimizing the industrial structure^44^. Fiscal and policy guidance measures of ESER demonstration cities have significant promotional effects on the upgrading of industrial structure^45^. In practice, the industrial structure upgrading effect is obvious in ESER demonstration cities, such as Urumqi city and Jilin city. The ratio of the added value of primary, secondary and tertiary industries in Urumqi was adjusted from 1.2:38.1:60.7 to 1.1:29.7:69.3, and the development of industrial clusters has been steadily advancing. The proportion of the added value of primary, secondary and tertiary industries in Jilin City was adjusted from 10:50.5:39.5 to 9.5:47.8:42.7, and advanced industries such as information service industry were vigorously developed.

On this basis, the upgrading of industrial structure will promote UGTI. On the one hand, as pilot cities need to accomplish ESER targets, they will attract more environmentally-friendly tertiary industries into their jurisdictions and promote the agglomeration of tertiary industries^46^. The tertiary industry not only has the characteristics of environmental protection, but also can better transform the achievements, which can produce more green patents, thus enhancing the level of UGTI. On the other hand, the strict environmental regulation policy is a barrier for pollution intensive enterprises to enter the market, and some secondary industry enterprises are eliminated. Therefore, the ratio of the tertiary industry over the secondary industry in the pilot cities has been improved^47,48^. Enterprises in the tertiary industry have a higher capacity for green technology innovation. The remaining secondary industry enterprises are subsidized by the environmental regulation to fundamentally reduce their costs through technological research and development and green innovation, thereby promoting UGTI. In addition, many empirical studies show that the upgrading of industrial structure, that is, the increase in the proportion of tertiary and secondary industries, is conducive to the promotion of UGTI^49,50^. For this reason, we propose the following hypothesis:

##### Hypothesis 2b

The ESER policy can induce UGTI by promoting industrial structure upgrading.

**Figure 1:**
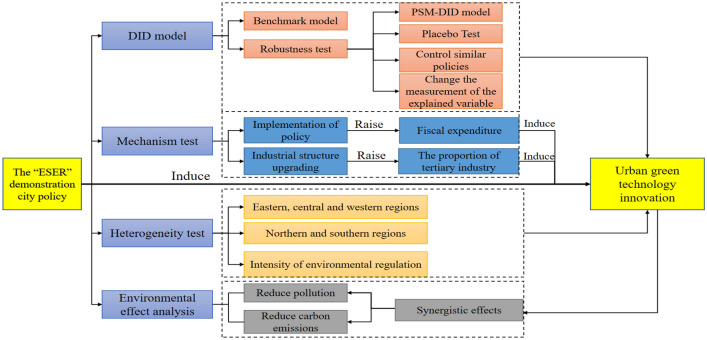
Theoretical analysis.

Figure 1 plots the theoretical analysis.

## Research design

### Identification strategy

To identify the effect of the ESER policy on UGTI, we consider the ESER demonstration city as a quasi-natural experiment, construct the DID model for testing, and set the benchmark model as follows:

1 $$ GINNO_{it} = \beta_{0} + \beta_{1} ESER_{it} + \beta_{2} X_{it} { + }\mu_{i} + \eta_{t} + \varepsilon_{it} $$

where the subscripts* i* and *t* represent the city and year, respectively; *GINNO* indicates UGTI, which is measured by the number of green patent applications per 10,000 people. *ESER* is the core explanatory variable, which refers to China’s ESER policy. *X* denotes the set of control variables. At the same time, *μ*_*i*_ and *η*_*t*_ represent the year fixed effects and the city fixed effects, respectively, and *ε*_*it*_ is the error term.

### Variable definition

#### UGTI

Green patents serve as a common metric for measuring *UGTI*, which can be categorized into two types: the number of green patent applications and the number of green patent grants. Since there is a certain time lag for patent grants, we choose the number of green patent applications to measure the level of *UGTI*^51,52^. Specifically, we take the logarithm of the number of green invention patent applications per 10,000 people (*GIP*) and the logarithm of the number of green utility model patent applications per 10,000 people (*GUP*) as the explained variables^53^.

#### The ESER demonstration city policy

**Figure 2:**
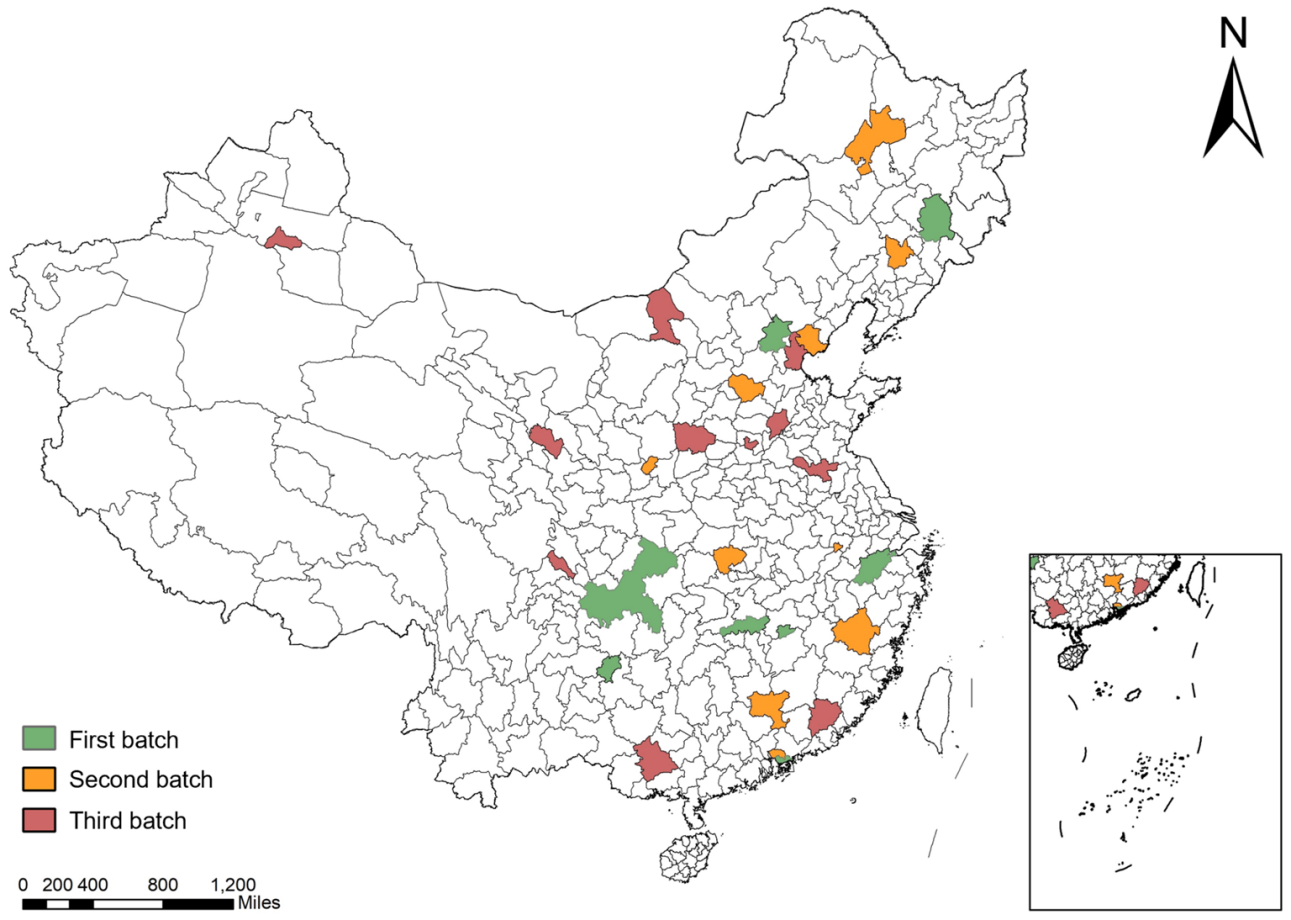
The distribution of ESER demonstration cities. *Note* the map was generated by ArcGIS Pro 10.2 software (https://www.esri.com/zh-cn/arcgis/products/arcgis-pro/trial).

We take the ESER policy implemented by the Chinese government in 2011 as the core explanatory variable. Among the 280 cities in our study sample, 30 cities are selected as pilot cities of ESER, which constitute our treatment group, while the remaining 250 cities constitute our control group. Figure 2 plots the spatial distribution of ESER demonstration cities. Specifically, the value of ESER in the selected year and the years after is 1 if a city is identified as a pilot city in ESER; otherwise, it takes the value of 0.

#### Control variables

To prevent the impact of omitted variables, we draw on literature and control the following city characteristic variables:

1. Economic growth (*GROWTH*): Cities with fast economic growth have strong financial strength, which can provide strong support for UGTI activities. Specifically, we use the real growth rate of GDP to measure the level of economic growth^54,55^.
2. Government intervention (*GOV*): The government can use incentives or penalties through the development of environmental regulations and financial subsidies, which can influence UGTI. Specifically, we use the ratio of fiscal general budget expenditure to GDP to represent government intervention^56^.
3. Population size (*POP*): On the one hand, population size affects the demand for energy and other resources; on the other hand, it brings technical knowledge, which has an impact on UGTI. Specifically, we use the natural logarithm of the urban population to measure population size^57^.
4. Human capital (*HC*): Human capital is not only the main body of technological research and innovation, but also has an impact on social green development through income level, consumption preference and environmental protection concepts to influence UGTI. Specifically, we use the number of college students per 10,000 people to represent the level of human capital^58^.
5. Financial development (*FIN*): Financial development affects the funding of innovation and research and development, which promotes UGTI, so financial development has a certain impact on UGTI. Specifically, we use the ratio of the deposit and loan balances of financial institutions to GDP to measure financial development^46^.
6. Foreign direct investment (*FDI*): *FDI* is closely linked to GTI, foreign technology spillovers from *FDI* affect UGTI, and developing countries will lower their environmental standards to attract *FDI*. Specifically, we use the ratio of total foreign direct investment to GDP to measure foreign direct investment^59^.
7. Infrastructure Construction (*ROAD*): Cities with good infrastructure are likely to attract more investment and talent inflow, which will have an impact on UGTI. Specifically, we use per capita road area to measure the level of infrastructure construction^60^.

### Data sources and descriptions

We use the panel data of 280 cities in China from 2006 to 2019 as the research object. The data are mainly obtained from the *China City Statistical Yearbook*, *China Urban Construction Statistical Yearbook* and the CEIC database. We obtain the green patent data from the Chinese Research Data Services (CNRDS) Platform, and the list of pilot cities of ESER is obtained by consulting relevant policy documents. The descriptive statistics of the variables are presented in Table 1.

**Table 1:** Descriptive statistics of the variables.

Variables	Variable definitions	Obs	Mean	Sd	Min	Max
*GIP*	Logarithm of the number of green invention patent applications per 10,000 people	3920	0.2663	0.4301	0.0000	3.3367
*GUP*	Logarithm of the number of green utility model patent applications per 10,000 people	3920	0.3054	0.4230	0.0000	3.2059
*ESER*	1 for pilot cities of ESER; 0 for non-pilot cities of ESER	3920	0.0531	0.2242	0.0000	1.0000
*GROWTH*	Real growth rate of GDP	3920	10.5778	4.3213	− 19.3800	32.9000
*GOV*	Ratio of fiscal general budget expenditure to GDP	3920	0.1787	0.0965	0.0426	1.4852
*POP*	Natural logarithm of the urban population	3920	5.8772	0.6944	2.8685	8.1362
*HC*	Number of college students per 10,000 people	3920	175.1077	231.5238	0.0000	1311.2407
*FIN*	Ratio of deposit and loan balances of financial institutions to GDP	3920	2.2265	1.1410	0.4369	21.3018
*FDI*	Ratio of total foreign direct investment to GDP	3920	0.0185	0.0193	0.0000	0.2101
*ROAD*	Per capital road area	3920	4.5211	5.8660	0.1812	73.0424

**Figure 3:**
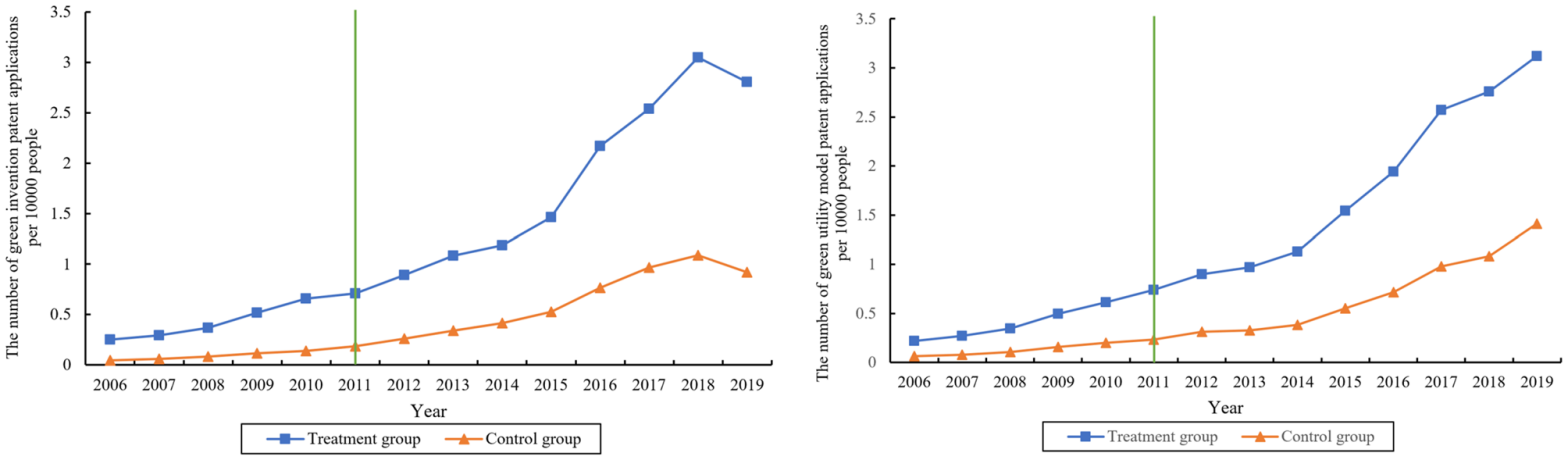
Changing trend of GIP and GUP.

Further, we analyze the changing trend of GIP and GUP in the treatment and control groups by graphically, which is shown in Fig. 3. We can see that the GIP and GUP of the two groups of cities show a rising trend during this period. After the official implementation of the ESER policy in 2011, the growth rate of GIP and GUP in the treatment group of cities accelerate significantly, and the gap with the control group of cities widened year by year. This may be due to the fact that the implementation of the ESER policy promotes the rapid increase of GIP and GUP, indicating an increase in the level of green technology innovation in the city. Of course, this descriptive analysis can’t exclude the influence of other factors such as time, which makes it difficult to accurately identify the net effect of the ESER policy. In the following, we will confirm this conclusion through DID model estimation.

## Empirical results and analysis

### Parallel trend test

Satisfying the parallel trend hypothesis, that is, before the implementation of the demonstration city of ESER, there is no systematic difference in the change trend of UGTI between ESER and non-ESER, which is the applicable premise of the DID model. Therefore, before estimating the benchmark regression model, we first use the event-study strategy to verify the parallel trend hypothesis. The specific model is set as follows:

2 $$ GINNO_{it} = \alpha_{0} + \sum\limits_{t = - 7}^{ - 1} {\beta_{t} \times before_{it} } + \sum\limits_{t = 0}^{8} {\beta_{t} \times after_{it} } + \varphi X_{it} + \mu_{i} + \eta_{t} + \varepsilon_{it} $$

where the core explanatory variables are *before*_*it*_ and *after*_*it*_, indicating the policy dummy variable from the year of implementation of the ESER pilot policy. Specifically, *before*_*it*_(*after*_*it*_) equals 1 if the year is in the t year before (after) the implementation of the policy, while it equals 0. The definitions of all other variables are the same as in Eq. (1). To avoid the impact of multicollinearity, we take the first eight years of policy implementation as the benchmark group (i. e. exclude the dummy variable of t = − 8) for estimation.

**Figure 4:**
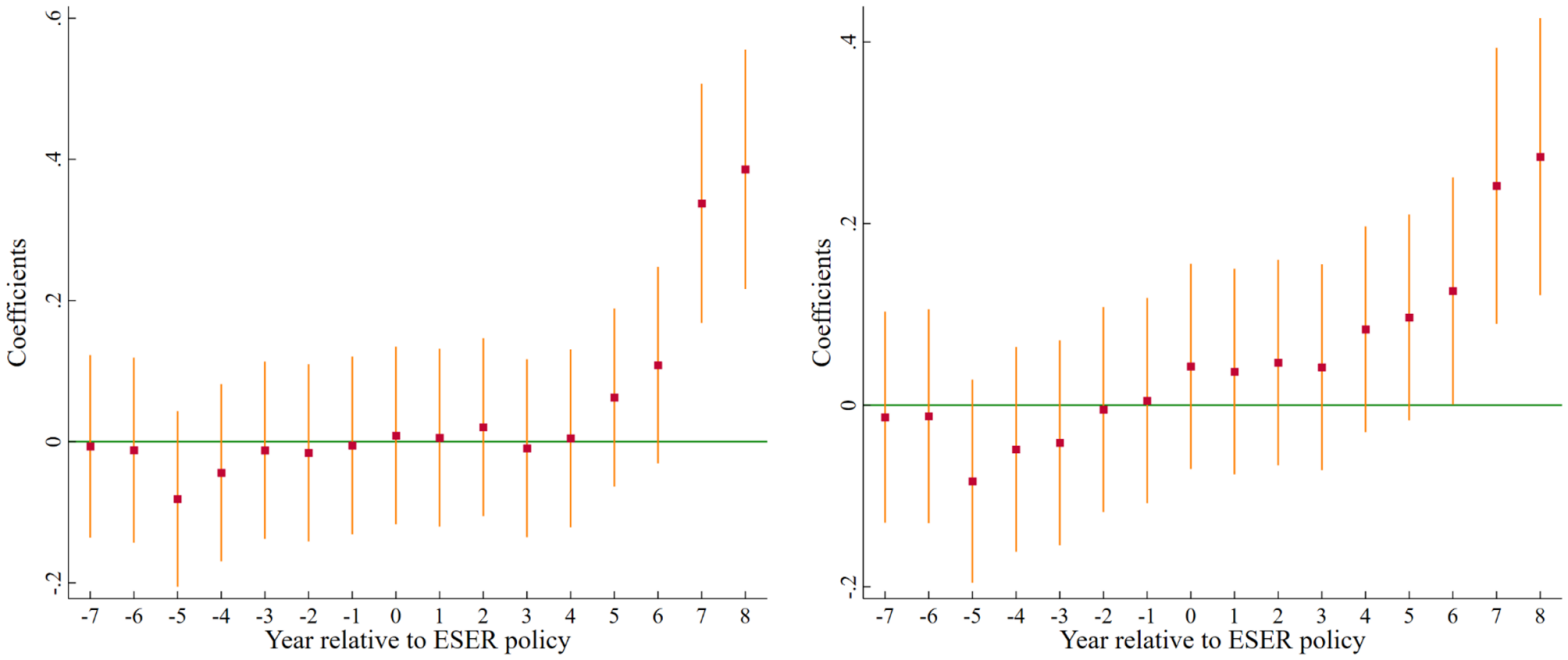
Parallel trend test of *GIP* (left) and *GUP* (right).

Figure 4 plots the regression coefficients of *before*_*it*_ and *after*_*it*_ and their 95% confidence intervals. It can be seen that the regression coefficients of *before*_*it*_ are all insignificant, indicating that there is no systematic difference in the trend of GTI between ESER and non-ESER before the implementation of the policy, thus supporting the parallel trend hypothesis.

Therefore, it is reasonable to use the DID model in our study. Meanwhile, the regression coefficient of *after*_*it*_ is positive but not significant in the year of policy implementation, and only starts to be significant after the 6th year of the policy implementation. This indicates that the implementation of ESER policy has a delayed impact on UGTI, which is the same as the conclusion supported by previous academics^61^. This is probably because even green patent applications also have a lag, and enterprises need to conduct research and development activities before patent applications, resulting in a longer lag in policy effects.

### Benchmark regression

The benchmark regression findings are shown in Table 2. Columns (1) and (3) show the results of the regression without the addition of any control variables, while columns (2) and (4) show the results of the regression after the addition of a number of control variables. All of the core explanatory coefficients are substantially positive, as seen in columns (1) through (4). These findings indicate that the ESER policy can significantly improve the level of UGTI. In terms of economic significance, the results in columns (2) and (4) show that compared to non-demonstration cities, the implementation of the ESER policy increases the number of green invention patents and utility model patents per 10,000 people in demonstration cities by 6.50% and 10.07%, respectively. In general, the policy can effectively improve UGTI, which provides evidence to support Hypothesis 1.

**Table 2:** Benchmark regression estimation results.

	(1)	(2)	(3)	(4)
	*GIP*	*GIP*	*GUP*	*GUP*
*ESER*	0.1309***	0.0650***	0.1560***	0.1007***
	(0.0209)	(0.0187)	(0.0190)	(0.0168)
*GROWTH*		0.0038***		0.0030***
		(0.0011)		(0.0010)
*GOV*		− 0.7328***		− 0.7224***
		(0.0790)		(0.0707)
*POP*		1.1782***		0.9053***
		(0.0567)		(0.0508)
*HC*		0.0007***		0.0006***
		(0.0001)		(0.0001)
*FIN*		0.0054		0.0098*
		(0.0062)		(0.0055)
*FDI*		− 2.2885***		− 2.5722***
		(0.2518)		(0.2254)
*ROAD*		0.0221***		0.0246***
		(0.0017)		(0.0015)
*Constant*	0.0549***	− 6.9000***	0.0677***	− 5.2883***
	(0.0119)	(0.3340)	(0.0107)	(0.2990)
City fixed effects	√	√	√	√
Year fixed effects	√	√	√	√
*N*	3920	3920	3920	3920
*R*^2^	0.4561	0.5785	0.5732	0.6765

*Note* Standard errors are in parentheses, ***, **, and * indicate significance at the 1%, 5%, and 10% level, respectively.

According to the estimated results of the control variables, the estimated coefficient of *GROWTH* is significantly positive at the 1% level, indicating that the economic growth of cities can provide financial support for UGTI, which in turn effectively promotes UGTI. The estimated coefficient of *GOV* is significantly negative at the 1% level, indicating that government intervention not only does not increase, but also hinders UGTI. The estimated coefficients of *POP* are 1.1782 and 0.9053, respectively, and are significantly positive at the 1% level, indicating that population size is an important factor in increasing UGTI. The estimated coefficients of *HC* and *ROAD* are significantly positive at the 1% level, indicating that high-quality human capital and infrastructure promote UGTI. The estimated coefficient of *FIN* is positive but insignificant, and *FDI* is significantly negative at the 1% level, indicating that *FDI* is not conducive to UGTI.

### Robustness tests

#### PSM-DID test

Cities with higher levels of economic development and a track record of success may be chosen as the demonstration cities for ESER before less developed cities, which could introduce “selective bias” into the results of the regression. Therefore, to overcome the impact of possible systematic bias between pilot cities and non-pilot cities on the regression results, we use the PSM-DID method to estimate the benchmark regression model. Specifically, we match the explanatory variables as outcome variables and the control variables as covariates using the least neighbor matching method.

**Figure 5:**
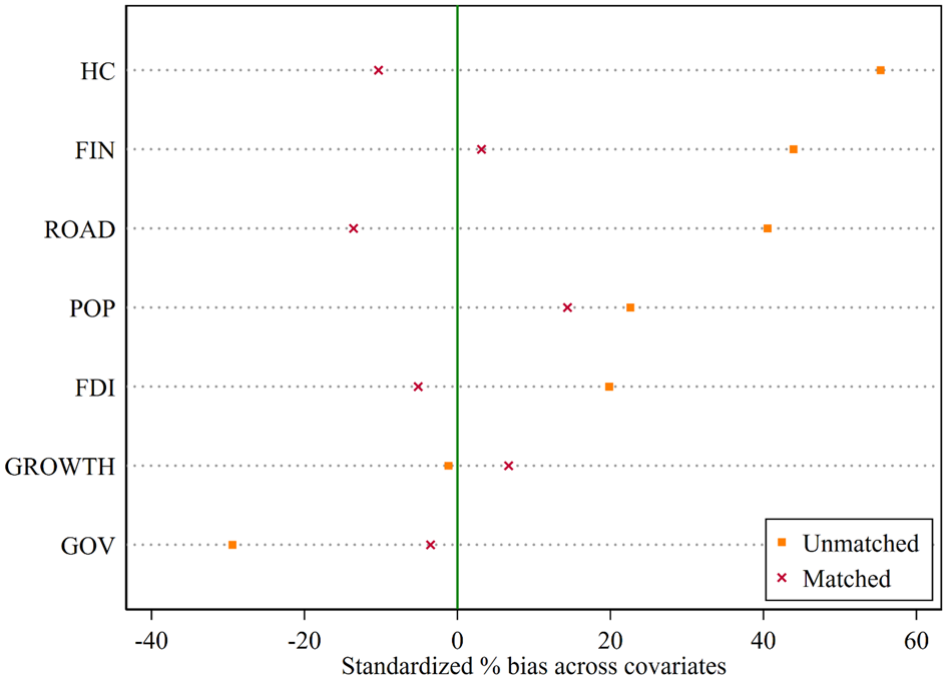
PSM balance test results.

The results of the PSM balance test are shown in Fig. 5. The absolute values of the standardized deviations of all covariates after matching are not more than 15%, indicating a good matching effect.

Based on the matched samples, we use the DID model for re-estimation. As shown in Table 3, after eliminating the self-selection errors and adding the control variables, urban and year fixed effects, the estimated coefficients of the core explanatory variable *ESER* for *GIP* and *GUP* are 0.0702 and 0.1012, respectively, and are still significantly positive at the level of 1%. The robust- ness of the regression results is further ensured and it shows that the ESER policy does induce UGTI.

**Table 3:** PSM-DID estimation results.

	(1)	(2)	(3)	(4)
	*GIP*	*GIP*	*GUP*	*GUP*
*ESER*	0.0978***	0.0702***	0.1214***	0.1012***
	(0.0201)	(0.0180)	(0.0184)	(0.0162)
Control variables		√		√
City fixed effects	√	√	√	√
Year fixed effects	√	√	√	√
*N*	3566	3566	3566	3566
*R*^2^	0.4616	0.5764	0.5774	0.6802

*Note* Standard errors are in parentheses, ***, **, and * indicate significance at the 1%, 5%, and 10% level, respectively.

#### Placebo test

**Figure 6:**
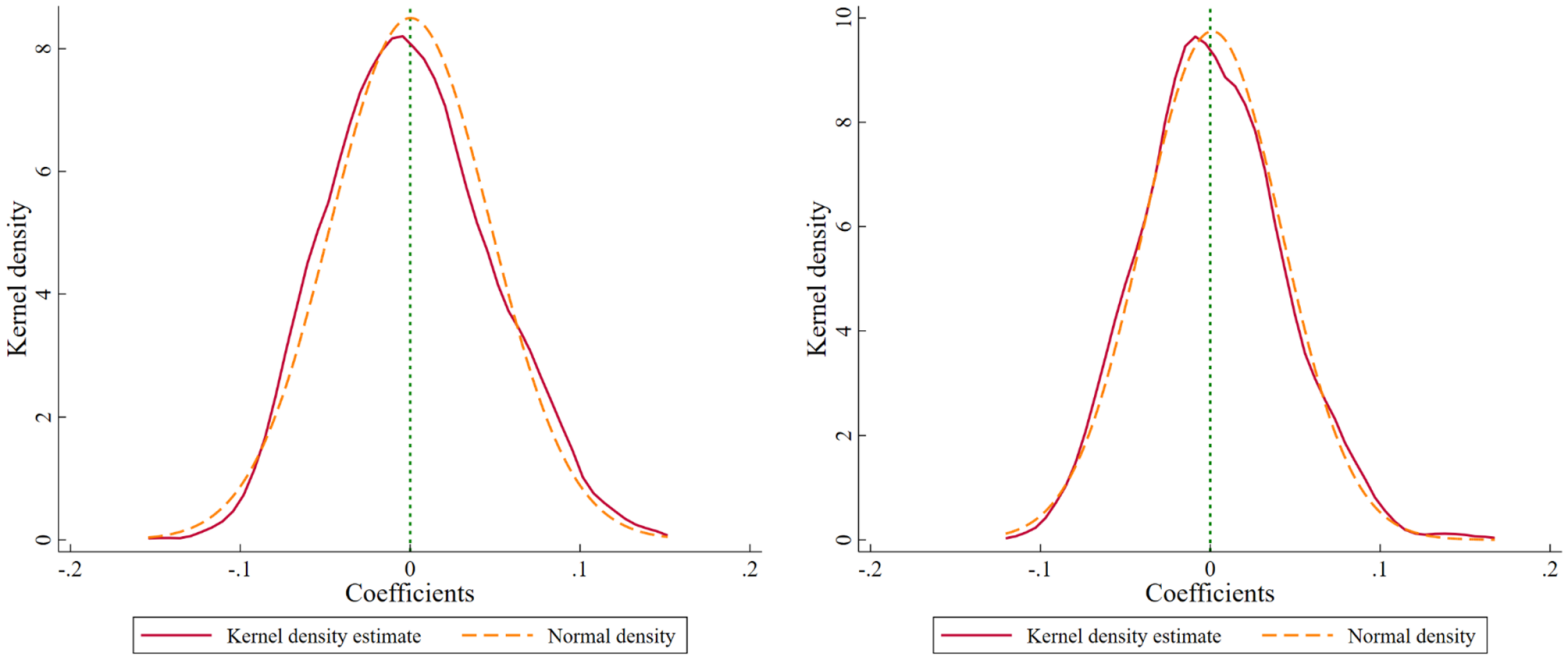
Placebo test of *GIP* (left) and *GUP* (right).

To exclude the influence of other random factors from influencing the estimation results, we construct a placebo test. In particular, 8, 10, and 12 cities are randomly chosen from the 280 cities in the study sample and used as a fictitious experimental group. The aforementioned procedure is performed 1000 times to obtain 1000 fictitious regression coefficients, assuming that these cities were founded as demonstration cities for ESER in 2011, 2013, and 2014, respectively. The kernel density of the erroneous regression coefficients is plotted in Fig. 6. As can be observed, the kernel density map is quite similar to the normal distribution, and the false regression coefficients of 1000 randomized trials are spread around 0. This indicates that the interference of other random factors fails to substantially affect the estimation results, providing further evidence that supporting the ESER policy can effectively induce UGTI.

#### Control other environmental policies

During the sample period, a number of other environmental policies were implemented in China, which may affect the level of UGTI and thus the accuracy of the estimation results in our study. Therefore, we need to reduce the impact of these policies to ensure the correctness of the outcomes of the ESER policy stimulating UGTI.

Specifically, we control the effects of two categories of policies: the first category is the carbon emissions trading pilot policy. China's National Development and Reform Commission chose seven provinces and cities in 2013 as pilot regions for carbon emissions trading rights, including Beijing, Tianjin, Shanghai, Chongqing, Shenzhen, Hubei Province, and Guangdong Province. The commission's goal is to combat climate change and control carbon emissions by changing the way the country's economy developed and modernizing its industrial structure^62^. The second is the pilot low-carbon city policy. To lower carbon emissions and create a safe and sustainable energy ecosystem, China's National Development and Reform Commission conducted three batches of low-carbon city pilot construction projects in 2010, 2012, and 2017^63^. The two types of policies mentioned above have the effect of increasing the level of green innovation in firms and cities^64^.

To eliminate the interference of the above policies, we create two dummy policy variables: *CARBON_TRADING* and *LOW_CARBON*. We add these two policy variables to the benchmark regression model. Table 4 presents the outcomes. The regression results are consistent with the estimated coefficients of *ESER* on *GIP* and *GUP*, which demonstrate the robustness of the benchmark regression results at the 1% level.

**Table 4:** Controlling the impact of other environmental policies.

	(1)	(2)	(3)	(4)	(5)	(6)
	*GIP*	*GIP*	*GIP*	*GUP*	*GUP*	*GUP*
*ESER*	0.0527***	0.0619***	0.0516***	0.0890***	0.0992***	0.0886***
	(0.0188)	(0.0187)	(0.0187)	(0.0168)	(0.0168)	(0.0168)
*CARBON_TRADING*	0.0985***		0.0884***	0.0942***		0.0908***
	(0.0168)		(0.0170)	(0.0150)		(0.0153)
*LOW_CARBON*		0.0503***	0.0393***		0.0246**	0.0133
		(0.0117)	(0.0119)		(0.0105)	(0.0106)
Control variables	√	√	√	√	√	√
City fixed effects	√	√	√	√	√	√
Year fixed effects	√	√	√	√	√	√
*N*	3920	3920	3920	3920	3920	3920
*R*^2^	0.5825	0.5807	0.5838	0.6800	0.6770	0.6801

*Note* Standard errors are in parentheses, ***, **, and * indicate significance at the 1%, 5%, and 10% level, respectively.

#### Replace the measurement of the explanatory variable

To avoid the influence of the selection of explanatory variables on the baseline regression results, according to the literature, we use the log of the number of green invention patents granted per capita (*GIPT*) and the log of the number of green utility model patents granted per capita (*GUPT*) as new explanatory variables in our study. The regression results are shown in Table 5. The results in columns (2) and (4) demonstrate that the estimated coefficient of *ESER* is significantly positive at the 1% level, which is the same as the baseline regression results, indicating that the conclusions are still valid after replacing the measurement of the explanatory variables.

**Table 5:** Replace the measurement of the explanatory variable.

	(1)	(2)	(3)	(4)
	*GIPT*	*GIPT*	*GUPT*	*GUPT*
*ESER*	0.0985***	0.0707***	0.1548***	0.1028***
	(0.0098)	(0.0089)	(0.0176)	(0.0157)
Control variables		√		√
City fixed effects	√	√	√	√
Year fixed effects	√	√	√	√
*N*	3920	3920	3920	3920
*R*^2^	0.3141	0.4515	0.5230	0.6325

*Note* Standard errors are in parentheses, ***, **, and * indicate significance at the 1%, 5%, and 10% level, respectively.

## Further analysis

### Mechanism analysis

To further explore the mechanism of the role of the ESER policy in improving UGTI, we construct the following mediation model for testing:

3 $$ GINNO_{it} = \beta_{0} + \beta_{1} ESER_{it} + \beta_{2} X_{it} { + }\mu_{i} + \eta_{t} + \varepsilon_{it} $$

4 $$ MED_{it} = \gamma_{0} + \gamma_{1} ESER_{it} + \gamma_{2} X_{it} { + }\mu_{i} + \eta_{t} + \varepsilon_{it} $$

5 $$ GINNO_{it} = \delta_{0} + \delta_{1} ESER_{it} + \delta_{2} MED_{it} + \delta_{3} X_{it} { + }\mu_{i} + \eta_{t} + \varepsilon_{it} $$

where *MED* represents the mediator variable, and the meaning of the remaining variables is consistent with Table 1. $\beta_{1}$ reflects the total effect of the ESER policy on UGTI. ${\gamma }_{1}$ shows the effect of the policy on mediating variable *MED*. ${\delta }_{2}$ is the effect of the mediating variable *MED* on UGTI after controlling the influence of the policy. ${\delta }_{1}$ is the direct effect of the policy on UGTI after controlling the effect of mediating variable *MED*.

According to Hypothesis 2a and Hypothesis 2b, we examine two mechanisms through which the policy promotes UGTI: fiscal science and technology expenditure and industrial structure upgrading, respectively. The corresponding mediator variables are set as follows: First, fiscal science and technology expenditure *(FIS*), which we measure by per capital fiscal science and technology expenditure^65^. Second, industrial structure upgrading (*INDUS*) is measured by the ratio of the value added of the tertiary industry to that of the secondary industry^66^.

#### Fiscal effect

Table 6 reports the results of the mediation effect model. According to the findings in column (1), the estimated coefficient of *ESER* is significantly positive at the 1% level, indicating that the implementation of the policy increases local government fiscal expenditures on science and technology. The results in columns (2) and (3) show that the estimated coefficient of *FIS* is also significantly positive at the 1% level, indicating that the increase in fiscal science and technology expenditure is conducive to the improving of UGTI level. At the same time, the estimated coefficients of (2) and (3) *ESER* are 0.0285 and 0.0672, smaller than the benchmark regression coefficients of 0.0650 and 0.1007, which means that in the process of promoting UGTI by the policy, fiscal science and technology expenditure play a partial intermediary role.

**Table 6:** Fiscal effect.

	(1)	(2)	(3)
	*FIS*	*GIP*	*GUP*
*ESER*	0.0120***	0.0285*	0.0672***
	(0.0027)	(0.0169)	(0.0150)
*FIS*		3.0443***	2.8045***
		(0.1034)	(0.0919)
Control variables	√	√	√
City fixed effects	√	√	√
Year fixed effects	√	√	√
*N*	3920	3920	3920
*R*^2^	0.2554	0.6600	0.7427

*Note* Standard errors are in parentheses, ***, **, and * indicate significance at the 1%, 5%, and 10% level, respectively.

#### Structural effect

The outcomes of the mediation effect model are presented in Table 7. According to the findings in column (1), the estimated *ESER* coefficient is notably positive at the 1% level, indicating that the implementation of the policy promotes the upgrading of urban industrial structure. The results in columns (2) and (3) show that the estimated coefficient of *INDUS* is significantly positive at the 1% level, indicating that the upgrading of urban industrial structure is conducive to the improvement of the UGTI level. Meanwhile, the estimated coefficients of *ESER* in columns (2) and (3) are 0.0602 and 0.0978, respectively, which are smaller than the benchmark regression coefficients of 0.0650 and 0.1007, and both are significant at the 1% level, indicating that industrial structure upgrading also plays a partial intermediary role in the process of promoting UGTI by the policy.

**Table 7:** Structural effect.

	(1)	(2)	(3)
	*INDUS*	*GIP*	*GUP*
*ESER*	0.0726***	0.0602***	0.0978***
	(0.0199)	(0.0187)	(0.0168)
*INDUS*		0.0657***	0.0396***
		(0.0156)	(0.0140)
Control variables	√	√	√
City fixed effects	√	√	√
Year fixed effects	√	√	√
*N*	3920	3920	3920
*R*^2^	0.5776	0.5806	0.6772

*Note* Standard errors are in parentheses, ***, **, and * indicate significance at the 1%, 5%, and 10% level, respectively.

### Heterogeneity analysis

#### East-center-west heterogeneity

Based on regional distinctions, we divide the sample into three groups: eastern, central and western cities, and estimate separately. When *GIP* is used as the explanatory variable, the coefficient of *ESER* is positive in all regions, but only significant in the western region, according to the regression results in Table 8. When *GUP* is used as the explanatory variable, the coefficient of *ESER* is significantly positive in different regions, and the western region has the largest coefficient. This indicates that the promotion effect of the policy on GTI in western cities is higher than that in other regional cities. This may be because western cities are more backward in economic development and their industrial structure is not advanced for the time being. Through the funds given by the ESER policy, the western cities effectively upgraded their industrial structure and fully mobilized the GTI initiative.

**Table 8:** Heterogeneity of urban geographical location.

	(1)	(2)	(3)	(4)	(5)	(6)
	Eastern	Central	Western	Eastern	Central	Western
	*GIP*	*GIP*	*GIP*	*GUP*	*GUP*	*GUP*
*ESER*	0.0337	0.0181	0.1001***	0.0681**	0.0413**	0.0997***
	(0.0342)	(0.0281)	(0.0259)	(0.0299)	(0.0204)	(0.0243)
Control variables	√	√	√	√	√	√
City fixed effects	√	√	√	√	√	√
Year fixed effects	√	√	√	√	√	√
*N*	1400	1386	1134	1400	1386	1134
*R*^2^	0.6541	0.5944	0.6103	0.7584	0.7433	0.7085

*Note* Standard errors are in parentheses, ***, **, and * indicate significance at the 1%, 5%, and 10% level, respectively.

#### North–south heterogeneity

In recent years, the widening gap between the north and the south in China has become increasingly prominent. As a result, we also investigate locational heterogeneity from the perspective of North–South differences. The regression results in Table 9 show that the coefficients of *ESER* are all positive when *GIP* is the explanatory variable, but are significant only in the northern region. When *GUP* is used as the explanatory variable, the coefficient of *ESER* is significantly positive in different regions, but the coefficient is larger in the northern region. This indicates that ESER has a higher contribution to UGTI in the north than in the south. This may be due to the development path dependence of high energy consumption and high carbon emissions in northern regions^67^. The policy requires cities in northern regions to reduce ESER, and financial subsidies can encourage enterprises to carry out green technology innovation activities and industrial upgrading, thus enhancing the level of UGTI.

**Table 9:** Heterogeneity of urban geographical location.

	(1)	(2)	(3)	(4)
	Southern	Northern	Southern	Northern
	*GIP*	*GIP*	*GUP*	*GUP*
*ESER*	0.0368	0.1116***	0.0799***	0.1368***
	(0.0303)	(0.0191)	(0.0271)	(0.0174)
Control variables	√	√	√	√
City fixed effects	√	√	√	√
Year fixed effects	√	√	√	√
*N*	2142	1778	2142	1778
*R*^2^	0.6108	0.5691	0.6692	0.7422

*Note* Standard errors are in parentheses, ***, **, and * indicate significance at the 1%, 5%, and 10% level, respectively.

#### Heterogeneity of government environmental attention

The green innovation effect of ESER policies may vary depending on government environmental attention. Therefore, we examine heterogeneity in terms of government environmental attention. Government environmental attention is represented by the frequency proportion of environment-related words in *The Government Work Report*. The samples are divided into strong and weak sub-samples based on the median of government environmental attention, and are estimated respectively.

The regression results in Table 10 show that the coefficients of *ESER* are positive when GIP is the explanatory variable, but only in cities with weak government environmental attention; when GUP is the explanatory variable, the coefficient of ESER is significantly positive in different regions, but the coefficient is larger in cities with government environmental attention. It shows that ESER policy has a greater effect on promoting UGTI with weak government environmental attention. This may be because current environmental regulation policies in China mainly target the pollution emissions of enterprises. In cities with weak government environmental attention, when the cost of pollution payment and acquisition of green technology is lower than green technology innovation, enterprises lack motivation and enthusiasm to carry out green technology innovation^68^. However, the policy not only has ESER requirements, which increase the cost of environmental pollution for enterprises but also has financial subsidies, which stimulate enterprises to carry out green technology innovation. Therefore, in cities with weak government environmental attention, the implementation of the policy makes enterprises tend to increase green technology innovation to obtain long-term benefits, and the policy plays a greater role in promoting UGTI.

**Table 10:** Heterogeneity of urban environmental regulation.

	(1)	(2)	(3)	(4)
	Strong	Weak	Strong	Weak
	*GIP*	*GIP*	*GUP*	*GUP*
*ESER*	0.0337	0.0476*	0.0558**	0.0884***
	(0.0288)	(0.0279)	(0.0258)	(0.0249)
Control variables	√	√	√	√
City fixed effects	√	√	√	√
Year fixed effects	√	√	√	√
*N*	1960	1960	1960	1960
*R*^2^	0.5824	0.5793	0.6683	0.6901

*Note* Standard errors are in parentheses, ***, **, and * indicate significance at the 1%, 5%, and 10% level, respectively.

### The impact of the 11th five-year plan

During the 11th Five-Year Plan, Chinese government set a target of reducing the country's total sulfur dioxide (SO_2_) emission by 10%. Taking into account factors such as environmental quality, environmental capacity, emission base and economic development level, almost all provinces have their own energy-saving and pollution-reducing targets. During this period, the central government linked the performance evaluation system for local officials to the energy intensity target. Success in achieving the energy intensity target will bring promotion opportunities for local officials^69^.

In order to test the impact of environmental targets set by provinces in the 11th Five-Year Plan on the green technology innovation effect of ESER policy, we refer to the research of Geng et al.^70^, and the following regression model is constructed:

6 $$ GINNO_{it} = \beta_{0} + \beta_{1} ESER_{it} + \beta_{2} ESER_{it} \times TARGET_{it} + \beta_{3} X_{it} { + }\mu_{i} + \eta_{t} + \varepsilon_{it} $$

*TARGET* represents the total SO_2_ emission control target of each province during the 11th Five-Year Plan period. The data comes from the “Reply of The State Council on the Total Emission Control Plan of Major Pollutants during the 11th Five-Year Plan Period”.

Table 11 reports the estimated results of model (6). The coefficients of *ESER* × *TARGET* are all significantly positive, indicating that the ESER policy has a stronger promoting effect on UGTI in cities where environmental targets are more stringent^71^.

**Table 11:** The impact of the eleventh five-year plan.

	(1)	(2)	(3)	(4)
	*GIP*	*GIP*	*GUP*	*GUP*
*ESER*	0.0380	− 0.0607	0.0559	− 0.0377
	(0.0452)	(0.0404)	(0.0409)	(0.0362)
*ESER* × *TARGET*	0.0084**	0.0113***	0.0091***	0.0125***
	(0.0036)	(0.0032)	(0.0033)	(0.0029)
Control variables		√		√
City fixed effects	√	√	√	√
Year fixed effects	√	√	√	√
*N*	3920	3920	3920	3920
*R*^2^	0.4569	0.5800	0.5741	0.6782

*Note* Standard errors are in parentheses, ***, **, and * indicate significance at the 1%, 5%, and 10% level, respectively.

### Environmental benefit analysis

To fulfill the dual carbon aim and prevent air pollution in China, which is an effective strategy to promote green and high-quality development, there is a concern about the synergistic reduction of pollution and carbon emissions^72^. Theoretically possible is the synergistic effect of concurrent pollution and carbon emissions reduction due to the same root, same source, same process aspect^73,74^. Reducing air pollution through green technology innovation while reducing carbon dioxide emissions, thus contributes to global climate change mitigation. Therefore, it is of great significance to study green technology innovation to achieve pollution reduction and carbon reduction.

To this end, we investigate whether the ESER policy can further promote the synergistic effect of pollution reduction and carbon reduction on the basis of promoting UGTI. The specific construction model is as follows:

7 $$ ENV_{it} = \beta_{0} + \beta_{1} GINNO_{it} + \beta_{2} X_{it} { + }\mu_{i} + \eta_{t} + \varepsilon_{it} $$

where $EN{V}_{it}$ serves as the explanatory variable and we measure the pollution reduction effect by the natural logarithm of SO_2_ emissions and the carbon reduction effect by the natural logarithm of CO_2_ emissions. The outcomes of the environmental benefit analysis are shown in Table 12. UGTI can successfully lower carbon emissions, as shown by the estimated coefficients of *GIP* in columns (1) and (2) being significantly negative at the 1% level and the estimated coefficient of *GUP* being significantly negative at the 10% level. The findings in columns (3) and (4) demonstrate that at the 1% level, the estimated coefficients of *GIP* and *GUP* are both notably negative, demonstrating that UGTI can greatly lower sulfur dioxide emissions. In summary, GTI has good environmental benefits.

**Table 12:** Environmental benefit analysis.

	(1)	(2)	(3)	(4)
	CO_2_	CO_2_	SO_2_	SO_2_
*GIP*	− 0.0818***		− 0.4033***	
	(0.0280)		(0.0451)	
*GUP*		− 0.0514*		− 0.4105***
		(0.0312)		(0.0503)
Control variables	√	√	√	√
City fixed effects	√	√	√	√
Year fixed effects	√	√	√	√
*N*	3920	3920	3920	3920
*R*^2^	0.3488	0.3478	0.6468	0.6455

*Note* Standard errors are in parentheses, ***, **, and * indicate significance at the 1%, 5%, and 10% level, respectively.

## Conclusions and policy implications

UGTI is a new engine for promoting the development of a green economy and is becoming the centerpiece of countries' eco-development strategies. It has become a matter of concern whether environmental regulation policies, such as the ESER policy, can induce UGTI. Based on the panel data of 280 Chinese cities during 2006–2019, this paper considers China’s ESER demonstration city policy as a quasi-natural experiment and uses the DID method to examine the policy’s effect and mechanisms on UGTI.

The results demonstrate that the ESER policy can effectively provide the level of UGTI, and this research conclusion still holds after a parallel trend test, placebo test and robustness test. Specifically, the policy reduces UGTI in two ways: increasing fiscal expenditures on science and technology and promoting industrial structure upgrading. In addition, the effect of the policy on UGTI is heterogeneous; the policy effect is more significant in the western region, the northern region, and cities with weak government environmental attention. At the same time, China’s ESER policy has a stronger promoting effect on UGTI in cities where environmental targets are more stringent. Moreover, the policy effectively induces UGTI, and UGTI has good environmental benefits of pollution and carbon reduction.

The research results discussed above have significant policy ramifications. First, the ESER policy can effectively raise the level of UGTI, and in the future economic development, the relevant departments will fully summarize the pilot experience brought by the policy, gradually broaden the scope and depth of the coverage of demonstration cities, promoting the continuous improvement of the level of UGTI. Second, the study demonstrates two ways in which the policy can raise the level of UGTI: increasing fiscal expenditures on science and technology and promoting industrial structure. To simultaneously accomplish economic growth, pollution reduction, and carbon reduction, the government should support the continual improvement of economic quality, raise fiscal spending, optimize the structure of fiscal expenditure, and support the upgrading of industrial structure. Finally, we consider the diversity of UGTI benefits brought about by the policy in various cities. Future policy design should adapt to local conditions so that the policy has a wider impact, especially in central, eastern, southern and cities with high levels of environmental regulation.

There are still some limitations in our study. First, there are still some errors in the measurement of UGTI. There is also some error in the number of green patent applications we use, and it is perhaps possible to use other measures of UGTI. In the future research, more indicators and methods should be used to measure UGTI to ensure the robustness of the research results. Second, we mainly consider ESER policy to promote UGTI from financial science and technology expenditures and industrial structure upgrading, in which there may be other mediating or moderating factors affecting the relationship between them. It can be further explored and improved in the future empirical analysis.

## Data Availability

The datasets used and/or analyzed during the current study available from the corresponding author on reasonable request.
